# VAR2CSA-reactive IgG in Brazilian women exposed to *Plasmodium falciparum* or *P. vivax* infection during pregnancy

**DOI:** 10.1128/iai.00516-25

**Published:** 2025-10-31

**Authors:** Maria Inês dos Santos, Jamille Gregório Dombrowski, Henriette Hoffmann-Veltung, Maria del Pilar Quintana, Claudio Romero Farias Marinho, Lars Hviid, Mary Lopez-Perez

**Affiliations:** 1 Department of Parasitology, Institute of Biomedical Sciences, University of São Paulo 28133, São Paulo, Brazil; 2 Centre for translational Medicine and Parasitology, Department of Immunology and Microbiology, Faculty of Health and Medical Sciences, University of Copenhagen 4321, Copenhagen, Denmark; 3 Department of Infectious Diseases, Rigshospitalet 53146, Copenhagen, Denmark; University of California Davis, Davis, California, USA

**Keywords:** Fc-afucosylation, immunity, immunoglobulin G (IgG), malaria, pregnancy-associated malaria, recombinant proteins, VAR2CSA

## Abstract

Placental malaria (PM) causes mortality and severe morbidity in areas with stable *Plasmodium falciparum* transmission. The selective placental accumulation of *P. falciparum*-infected erythrocytes (IEs) is mediated by VAR2CSA, a PfEMP1-type parasite ligand that binds exclusively to placenta-restricted CSA. VAR2CSA-specific IgG is therefore generally restricted to women exposed to *P. falciparum* infection during pregnancy. However, widespread acquisition of VAR2CSA-reactive IgG outside pregnancy among Colombian and Brazilian individuals has been reported, supposedly due to cross-reactivity between VAR2CSA and the *P. vivax*-specific antigen PvDBP. Here, we measured levels and Fc-afucosylation of VAR2CSA-reactive IgG in plasma from pregnant Brazilian women at delivery, using full-length VAR2CSA (FV2) expressed in baculovirus-transfected insect cells (FV2_BIC_) or Chinese hamster ovary cells (FV2_CHO_) as well as the corresponding native antigen (IT4VAR04) on the IE surface. We also measured levels of IgG specific for GLURP (*P. falciparum-*specific) and PvDBP (*P. vivax*-specific). FV2_CHO_-specific IgG levels were lower than FV2_BIC_-reactive IgG levels. Furthermore, only FV2_CHO_-specific IgG was restricted to women exposed to *P. falciparum* during pregnancy. Levels of PvDBP-specific IgG were significantly higher among *P. vivax*-exposed pregnant women but did not correlate with FV2-specific IgG levels. Finally, FV2_CHO_-specific IgG was markedly Fc-afucosylated in contrast to FV2_BIC_-reactive IgG. Our findings caution against using levels of IgG reacting with recombinant proteins expressed in insect cells as a measure of exposure to VAR2CSA during pregnancy, at least in South America. Furthermore, our data do not support the hypothesis that exposure to PvDBP induces IgG that cross-reacts with VAR2CSA and contributes to protection against PM.

## INTRODUCTION

In 2023, there were an estimated 263 million cases of malaria, of which about 597,000 were fatal (1). Most severe cases of malaria, and essentially all fatal ones, are caused by infection with *Plasmodium falciparum* and are highly concentrated among young children and pregnant women in tropical Africa. Placental malaria (PM) is a major contributor to that distribution of morbidity and mortality (2). PM is caused by selective accumulation of infected erythrocytes (IEs) in the placenta, mediated by *P. falciparum* expressing a particular type (VAR2CSA) of the PfEMP1 antigens that the parasites employ to make IEs adhere in the vasculature and thus avoid destruction in the spleen (3, 4). Expression of VAR2CSA is detrimental to parasites in non-pregnant hosts, as the host receptor for VAR2CSA (chondroitin sulfate A; CSA) is normally only expressed by the placental syncytiotrophoblast and therefore not available outside pregnancy. Indeed, IEs infecting non-pregnant hosts do not adhere to CSA and do not express VAR2CSA. Consistent with this, children, men, and women who have never been pregnant possess little or no VAR2CSA-specific IgG (5–8). By adolescence, clinical disease is rare in highly endemic areas, whereas low-density, asymptomatic infections that can last for years without reinfection remain prevalent (9, 10). PM often originates from such infections that are present at conception but which have been controlled to very low densities by IgG targeting antigens other than VAR2CSA (11–13). However, switching to expression of VAR2CSA when placental CSA appears after conception confers VAR2CSA^+^ IEs with a major survival advantage, particularly in primigravidae. Indeed, parasites transcribing *var2csa* as well as VAR2CSA-specific IgG start appearing early in pregnancy (14, 15). This etiology compromises the efficiency of PM interventions designed to prevent new infections, including insecticide-treated bed nets and currently available malaria vaccines (16, 17). Vaccines based on VAR2CSA and designed specifically to prevent PM are therefore in development (18, 19). In addition, it has been suggested that epitopes in the *P. vivax*-specific antigen PvDBP cross-react with epitopes in VAR2CSA (20), allowing acquisition of immunity to PM outside pregnancy and in areas where *P. falciparum* is rare but *P. vivax* is common, such as South America (21). This putative cross-species immune response is controversial (22–24). However, if it were genuine, it might enable a completely novel approach to vaccination against PM, employing a PvDBP-based antigen (20). To further inform efforts to develop PM-specific vaccines, we characterized IgG responses to VAR2CSA, PvDBP, and additional *P. falciparum*- and *P. vivax*-specific antigens in a cohort of Brazilian pregnant women.

## MATERIALS AND METHODS

### Study population, samples, and data

The samples used here originated from a cohort/case-control study of malaria in pregnancy, conducted in the Juruá Valley region, Acre state, Brazil (25, 26). Clinical and epidemiological data were collected from study participants at three home visits during pregnancy and at delivery. Infection with *P. falciparum* and *P. vivax* was assessed on each occasion by microscopy of thick and thin peripheral blood smears and confirmed by real-time quantitative PCR. All women who were infected with malaria parasites were treated according to the Brazilian Ministry of Health guidelines (25). All available time-of-delivery plasma samples (*N* = 380) from the original study (after exclusion of women infected with both *P. falciparum* and *P. vivax*) were included in the present study. The clinical characteristics of the sample donors are summarized in Table 1.

**TABLE 1:** Clinical characteristics of study participants

Characteristic	*P. falciparum* (*N* = 70)	*P. vivax* (*N* = 150)	Non-infected (*N* = 160)	All (*N* = 380)	*P* value
Age in years^*a*^	22 [19–24]	21 [19–23]	24 [22–25]	22 [21–23]	0.01^*b*^
Gestational age in weeks^*a*^	39 [39–40]	39 [39–40]	40 [39–40]	40 [39–40]	0.17^*b*^
Primigravidae	26 (37.1%)	64 (42.7%)	76 (47.5%)	47 (12.4%)	0.53^*c*^

^*a*^ Median and 95% confidence intervals.

^*b*^ Kruskal-Wallis test.

^*c*^ Chi-square test.

Pooled plasma from anonymous *P. falciparum-*exposed pregnant women from Ghana and individual samples from 17 adult Danish donors never exposed to *P. falciparum* infection were included as positive and negative controls, respectively.

### Recombinant parasite proteins

The entire ectodomain of the VAR2CSA-type PfEMP1 protein IT4VAR04 (GenBank accession number GU249598; encoding amino acids Met1 to Phe2649) was codon-optimized and cloned into appropriate vectors with C-terminal V5 and histidine tags (FV2_BIC_) or an SG4S linker followed by a histidine tag (FV2_CHO_). The proteins were produced in baculovirus-transfected insect cells (FV2_BIC_) and in Chinese hamster ovary cells (FV2_CHO_) as described (22, 27), except for an extra step of FV2_CHO_ purification by size exclusion chromatography (SEC) on a HiPrep 16/60 Sephacryl S-300 HR Column (Cytiva). The non-repetitive amino-terminal region (R0) of *P. falciparum* glutamate-rich protein (GLURP) (28) and the *P. vivax* Duffy- binding protein (PvDBP) were produced in *Escherichia coli* (29).

### Enzyme-linked immunosorbent assays (ELISA)

Plasma levels of IgG were measured in duplicate by ELISA as described (22) and expressed as arbitrary units (AU) using the average optical density (OD), calculated as 100 × [(OD_SAMPLE_ – OD_BLANK_)/(OD_POSCTRL_ – OD_BLANK_)]. Negative cutoffs were calculated as the mean AU plus three standard deviations of values obtained with the negative control donors. Assays were repeated in case of variation >20% between the duplicates. To normalize data between assays, positive and negative controls were included on all plates.

Fc-afucosylation of FV2_BIC_-reactive IgG was determined using a fucose-sensitive enzyme-linked immunosorbent (FEASI) assay method as described (30) and validated by us for use with VAR2CSA (31). FEASI involves two ELISAs, one to quantify antigen-specific IgG levels and a second to test its affinity for FcγRIIIa. The second ELISA was only run for plasma samples with FV2_BIC_-reactive IgG levels above the negative cutoff in the first ELISA. Briefly, 96-well flat-bottomed microtiter plates (Nunc MaxiSorp) were coated with recombinant full-length VAR2CSA (1 µg/mL) and incubated with four twofold serial dilutions of plasma. Bound IgG was detected with horseradish peroxidase (HRP)-conjugated anti-human IgG (Dako; 1 µg/mL) (first ELISA) or with biotinylated FcγRIIIa (R&D; 0.1 µg/mL), followed by streptavidin-poly-HRP (Sanquin; 0.05 µg/mL) (second ELISA), as detailed previously (30). Specific IgG levels were calculated and expressed as AU using a serially diluted Ghanaian plasma sample (set at 100 AU) as calibrator. Ratios of AU values from the two ELISAs were used to estimate the percentage of VAR2CSA-specific IgG fucosylation according to a simple linear regression as described (30), using samples with known fucosylation levels obtained by LC-MS. Calculated Fc-afucosylation values ≤0% or ≥100% were assigned values of 1% and 99%, respectively.

### Parasite cultures and flow cytometry

The *P. falciparum* clone IT4 was maintained in serum-free RPMI-1640 medium as described (32). Late-stage IEs were selected for surface expression of the VAR2CSA-type PfEMP1 of this clone (IT4VAR04) by immunomagnetic selection as described (33), using protein A-coupled DynaBeads coated with the VAR2CSA-specific human monoclonal antibody PAM1.4 (34). Plasma IgG reactivity with PAM1.4-selected IEs was analyzed by flow cytometry as described (33). In brief, late-stage IEs were labeled with plasma samples (1:20) followed by FITC-conjugated rabbit anti-human IgG (1:150; Jackson). Parasite nuclei were stained with Hoechst 33342 (10 μg/mL; Invitrogen). About 20,000 IEs were acquired by flow cytometry (CytoFLEX S, Beckman Coulter Life Sciences) and analyzed using FlowLogic software v. 8.3 (Inivai Technologies; Fig. S1). Median fluorescence intensity (MFI) of FITC for both noninfected (Hoechst-negative cells) and infected (Hoechst-positive cells) erythrocytes was calculated and used to report a normalized MFI (nMFI = MFIpos – MFIneg).

### Statistical analysis

Data were analyzed in GraphPad Prism v.10.5 (GraphPad Software). All experiments were repeated at least two times with similar results, and the average was used for the final analysis. Sample sizes and specific statistical tests are indicated in the text, figures, and legends. Two-tailed *P* values < 0.05 were considered statistically significant.

## RESULTS

### Levels of FV2_BIC_- and FV2_CHO_-reactive IgG are discordant in plasma from Brazilian pregnant women

**Fig 1:**
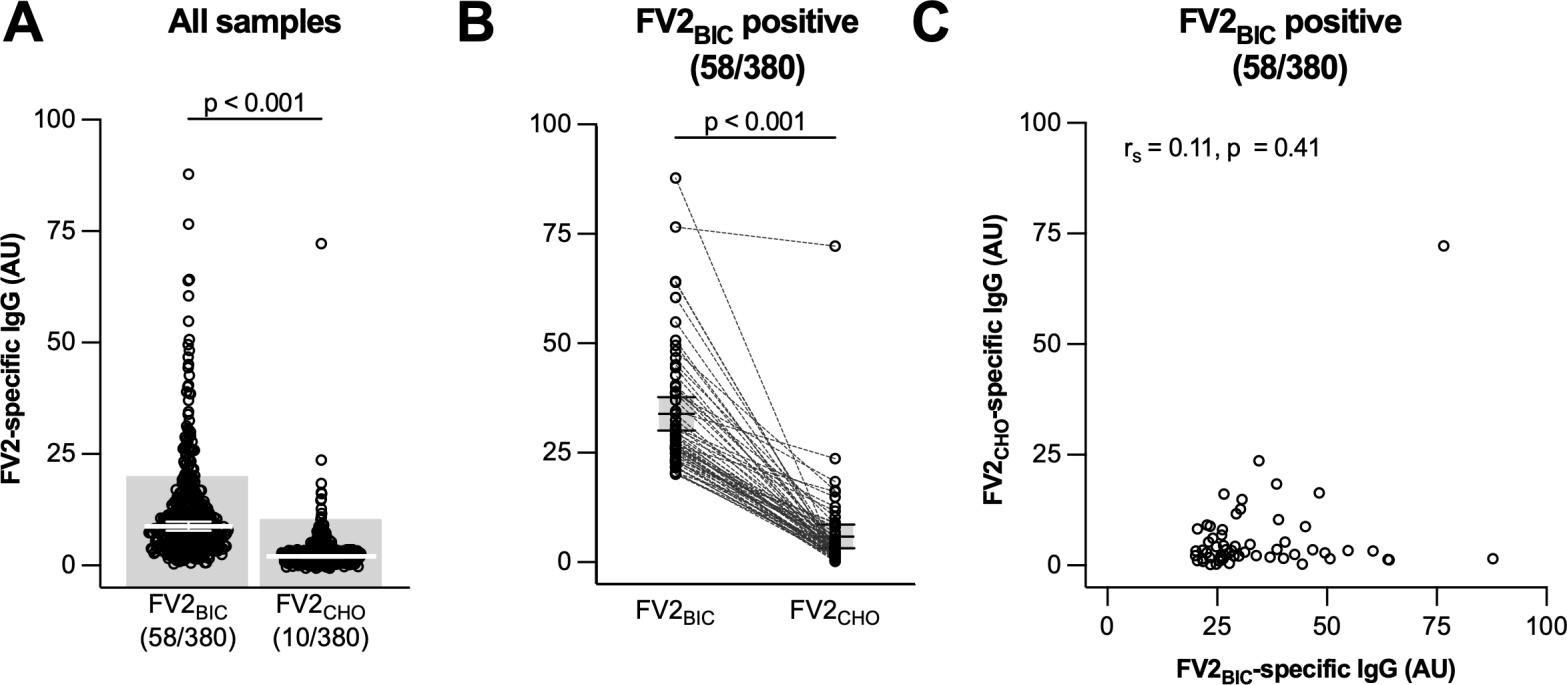
Antibody responses to full-length recombinant VAR2CSA proteins (FV2) in pregnant Brazilian women at delivery. (**A**) Responses to the full ectodomain of the VAR2CSA-type PfEMP1 antigen IT4VAR04 (FV2) expressed in baculovirus-infected insect cells (FV2_BIC_) and in CHO cells (FV2_CHO_). The number of data points above the negative cutoff is displayed, followed by the total number of data points. (**B**) Comparison of FV2_BIC_ and FV2_CHO_ responses in women with FV2_BIC_ responses above the negative cutoff. (**C**) Correlation between FV2_BIC_ and FV2_CHO_ responses in women with FV2_BIC_ responses above the negative cutoff. Individual data points, medians and 95% confidence intervals (panels **A** and **B** only), negative cutoff (shading, panel** A **only) and results of Wilcoxon signed-rank tests (**A and B**), and Spearman’s rank correlation (**C**) are shown.

Consistent with our earlier study from Colombia (22), levels of FV2_BIC_-reactive IgG above negative cutoff (AU = 20.1) were prevalent among Brazilian women at delivery (58/380, 15.3%). In contrast, levels of FV2_CHO_-specific IgG above negative cutoff (AU = 10.4) were infrequent (10/380, 2.6%) (Fig. 1A), despite having an VAR2CSA-encoding amino acid sequence identical to that of FV2_BIC_. Furthermore, in plasma samples with levels of FV2_BIC_-reactive IgG above negative cutoff, the corresponding levels of FV2_CHO_-specific IgG were significantly lower (Fig. 1B). Finally, although levels of FV2_BIC_- and FV2_CHO_-specific IgG correlated significantly (*r*_s_ = 0.42, 95% confidence interval [0.33–0.50], *P* < 0.001, *N* = 380), the correlation was weaker and non-significant if only the 58 samples with FV2_BIC_-reactive IgG above negative cutoff were considered (*r*_s_ = 0.11 [0.16–0.36], *P* = 0.41) (Fig. 1C). It thus appears that the Brazilian plasma samples studied here, like the Colombian samples we studied previously (22), contain substantial amounts of IgG reacting with FV2_BIC_ but not with FV2_CHO_.

### Levels of FV2_BIC_-reactive IgG in plasma from Brazilian pregnant women do not reflect IgG exposure to native VAR2CSA

**Fig 2:**
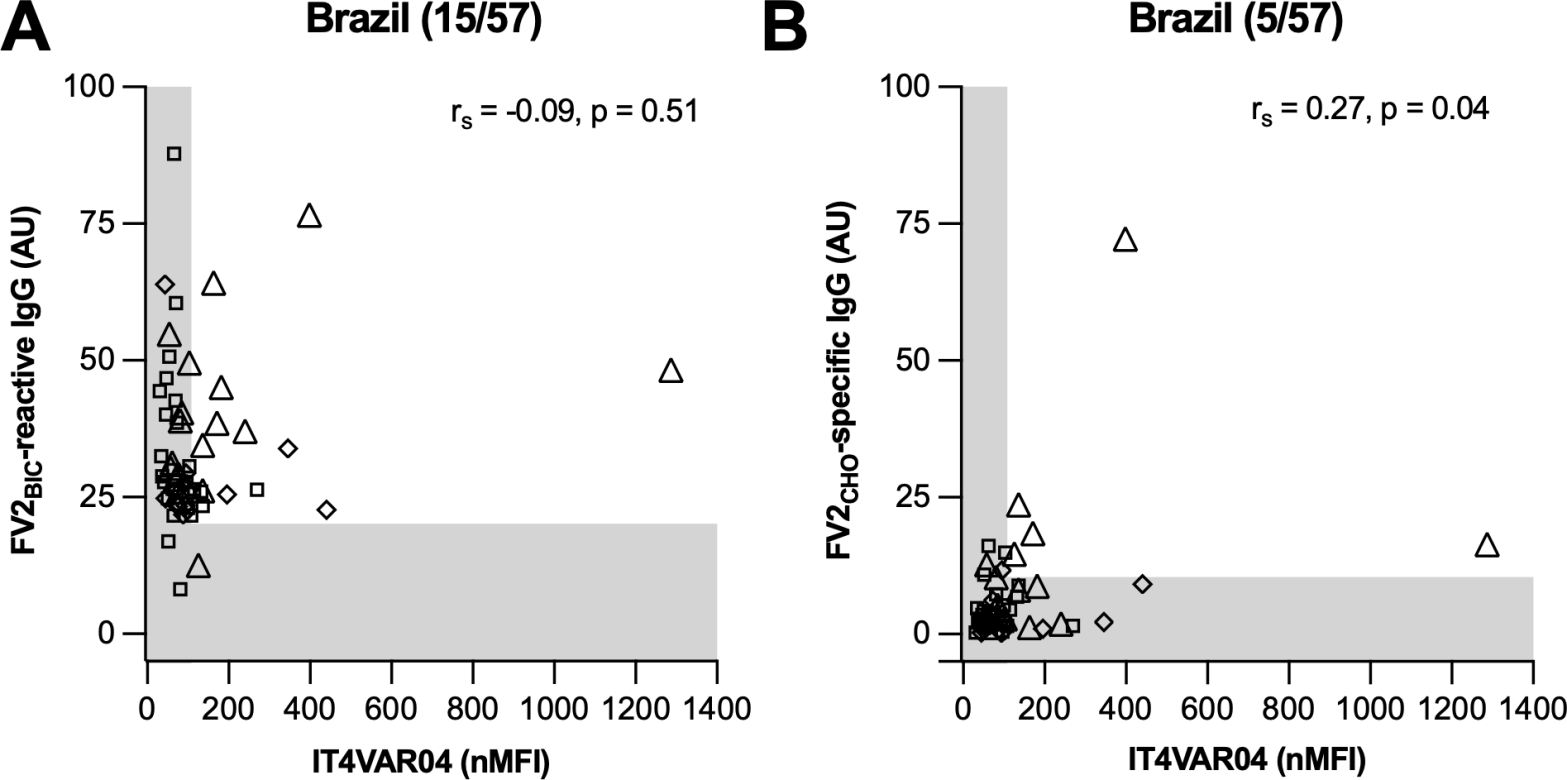
IgG responses to native VAR2CSA (IT4VAR04; normalized median fluorescence, nMFI) and FV2_BIC_ (**A**) or FV2_CHO_ (**B**) (arbitrary units) at delivery among Brazilian women exposed to *P. falciparum* (triangles), *P. vivax* (squares), or without evidence of infection (diamonds) during the current pregnancy. Individual data points, negative cutoffs (shading), Spearman’s rank correlations, and the number of data points above both negative cutoffs and the total number of data points (in brackets) are shown.

We have previously suggested that much of the IgG in Colombian individuals that reacts with recombinant proteins expressed in baculovirus-infected insect cells reacts with epitopes that are not present in native *P. falciparum* proteins and do not accurately reflect exposure to malaria parasites (22). To test this hypothesis, we assessed the ability of plasma from 57 Brazilian women with levels of FV2_BIC_-reactive IgG above negative cutoff to label IEs selected to express IT4VAR04 (the native VAR2CSA variant corresponding to FV2) on their surface. In agreement with our previous study from Colombia, there was no significant correlation between IgG reactivity with FV2_BIC_ and with IT4VAR04^+^ IEs (Fig. 2A). In contrast, IgG reactivity with FV2_CHO_ and with IT4VAR04^+^ IEs in the same samples correlated significantly (Fig. 2B). We conclude that levels of FV2_BIC_-reactive IgG in plasma from Brazilian pregnant women do not reliably reflect exposure to native VAR2CSA on the surface of IEs.

### Levels of FV2_BIC_-reactive IgG in plasma from Brazilian pregnant women do not reflect infection with *P. falciparum* or *P. vivax *during pregnancy

**Fig 3:**
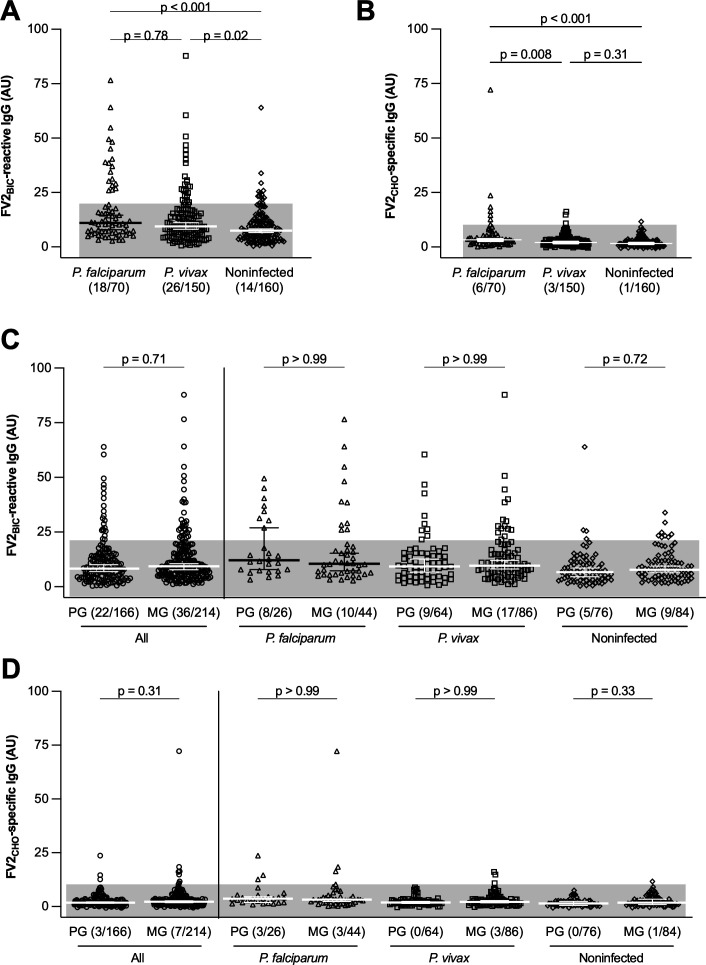
Exposure to malaria parasites during pregnancy and IgG responses to FV2 at delivery. FV2_BIC_-reactive IgG (**A**) and FV2_CHO_-specific IgG (**B**) among women with documented *P. falciparum* (triangle symbols) or *P. vivax* infection (square symbols) during the current pregnancy or without evidence of infection (noninfected, diamond symbols). (**C and D**) IgG responses by gravidity (primigravidae [PG] and multigravidae [MG]). Individual data points, medians, 95% confidence intervals, negative cutoff (shading), and results of Kruskal-Wallis test, followed by Dunn’s test for pairwise differences are shown. The number of data points above the negative cutoff is displayed, followed by the total number of data points.

**Fig 4:**
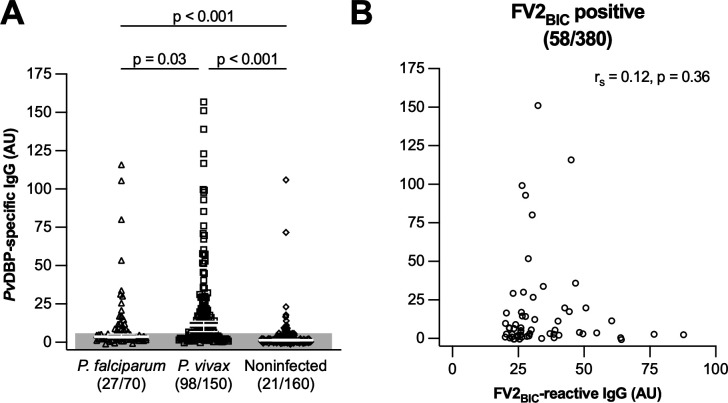
Antibody responses to PvDBP. (**A**) Exposure to malaria parasites during pregnancy and IgG responses to PvDBP at delivery among women with documented *P. falciparum* (triangles) or *P. vivax* infection (squares) or without evidence of infection (diamonds) during the current pregnancy. Individual data points, medians, 95% confidence intervals, negative cutoff (shading), and results of Kruskal-Wallis test, followed by Dunn’s test are shown. The number of data points above the negative cutoff is displayed, followed by the total number of data points. (**B**) Correlation between FV2_BIC_-reactive and PvDBP-specific IgG. Individual data points and Spearman’s rank correlations are shown.

*P. vivax* is the most prevalent species of malaria parasites in Colombia and Brazil, but it is rare in most African settings (1). This is due to the widespread absence in Africa of the Duffy antigen (35), which is required for erythrocyte invasion by *P. vivax* but not by *P. falciparum* (36, 37). To test the hypothesis that epitopes in the *P. vivax-*specific merozoite antigen PvDBP cross-react with epitopes in the *P. falciparum*-specific IE surface antigen VAR2CSA (38), we compared antigen-specific IgG levels among uninfected women and those infected by either *P. falciparum* or *P. vivax* during their current pregnancy (Table 1). As reported previously (22, 38), FV2_BIC_-reactive IgG above negative cutoff was found in all exposure groups (Fig. 3). There was no significant difference between the reactivity of women exposed to *P. falciparum* and *P. vivax* while pregnant, while reactivity was significantly less common among women without documented exposure to either parasite (Fig. 3A). In contrast, FV2_CHO_-specific IgG above negative cutoff was largely confined to women with documented *P. falciparum* infection during the current pregnancy and the median level was significantly higher than for *P. vivax*-exposed women (Fig. 3B). Furthermore, levels of FV2_CHO_-specific IgG among women with documented *P. vivax* exposure were not significantly different from levels among women without evidence of exposure to malaria parasites while pregnant (Fig. 3B). Finally, levels of neither FV2_BIC_-reactive (Fig. 3C) nor FV2_CHO_-specific IgG (Fig. 3D) differed between primigravidae and multigravidae, regardless of infection history. We conclude that levels of FV2_BIC_-reactive IgG in plasma from Brazilian pregnant women do not reliably reflect recent exposure to either *P. falciparum* or *P. vivax*. As independent measures of exposure to these two species of malaria parasites, we measured levels of IgG reacting with the *P. falciparum*-specific antigen GLURP (Fig. S2) and the *P. vivax*-specific antigen PvDBP (Fig. 4A). Reactivity to both antigens was common, with levels of GLURP- (Fig. S2) and PvDBP-specific IgG (Fig. 4A) reflecting recent exposure to *P. falciparum* and *P. vivax*, respectively.

### Levels of FV2_BIC_-reactive IgG in plasma from Brazilian pregnant women do not correlate with levels of PvDBP-specific IgG

According to the hypothesis regarding cross-reactivity between epitopes in PvDBP and VAR2CSA (38), levels of IgG reactive with these two antigens would be expected to correlate significantly. However, plasma levels of FV2_BIC_-reactive IgG in the Brazilian pregnant women correlated only weakly with levels of PvDBP-specific IgG, regardless of whether all the women were considered or they were divided according to parasite exposure (Table S1). If we considered only the 58 individuals with FV2_BIC_-reactive IgG levels above cutoff, the correlation between FV2_BIC_ and PvDBP IgG responses dropped further and was no longer statistically significant (Fig. 4; Table S1). Among South American individuals, IgG reactivity to recombinant antigens expressed in baculovirus-infected insect cells thus appears to be largely platform-dependent and not reflecting *P. falciparum-* (or *P. vivax*-) exposure.

### FV2_CHO_-specific, but not FV2_BIC_-reactive, IgG is selectively Fc-afucosylated

**Fig 5:**
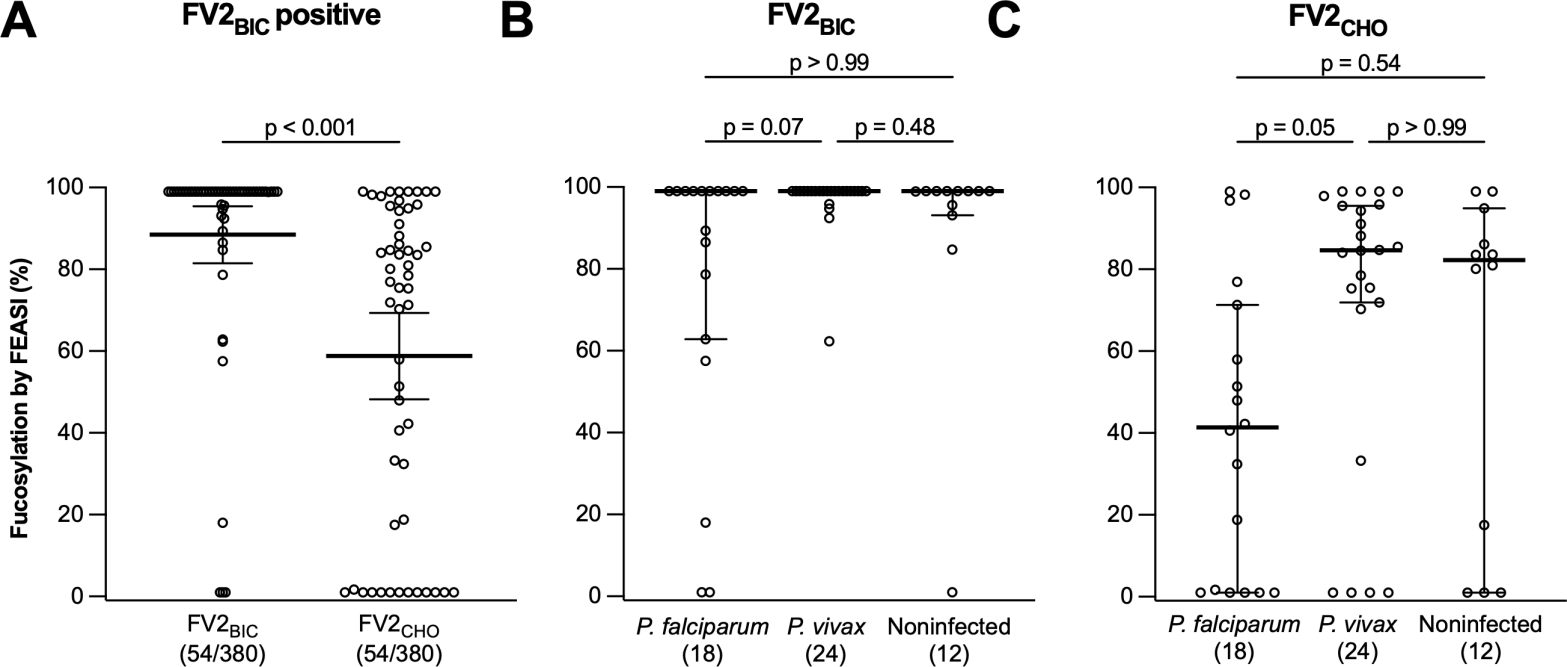
Fc-fucosylation of VAR2CSA-specific IgG (women with FV2_BIC_-reactive IgG above negative cutoff only). FV2_BIC_-reactive versus FV2_CHO_-specific IgG (**A**). FV2_BIC_-reactive (**B**) and FV2_CHO_-specific IgG (**C**) according to infection status (current pregnancy). Percentage of Fc-fucosylated IgG was estimated using FcγRIIIa/IgG ratio. Individual data points, medians, and their 95% confidence intervals, and results of Wilcoxon signed-rank test (**A**) and Kruskal-Wallis test followed by Dunn’s test (**B and C**) are shown. Sample size is shown in parentheses.

IgG is generally fully Fc-fucosylated at the single glycosylation site at position Asn297 in the Fc region of IgG (39). However, allo-IgG with specificity for Rhesus antigen and human platelet antigen 1a can be markedly Fc-afucosylated (40, 41). Less profound reductions in Fc-fucosylation have been observed for IgG specific for several enveloped virus antigens (42–44). It has been proposed that the post-translational modification required for secretion of Fc-afucosylated IgG is induced exclusively in B cells that recognize foreign antigens expressed on the surface of self-membranes (44). Consistent with this hypothesis, we have previously reported that VAR2CSA-specific IgG acquired in response to *P. falciparum* infection during pregnancy is selectively and markedly Fc-afucosylated (31, 45), whereas VAR2CSA-specific IgG induced by subunit vaccination is fully Fc-fucosylated (45). On this basis, we estimated the degree of Fc-afucosylation of FV2_BIC_- and FV2_CHO_-specific IgG in a subset of the plasma samples from Brazilian women. FV2_BIC_-reactive IgG was mostly fully Fc-fucosylated, whereas FV2_CHO_-specific IgG was often markedly Fc-afucosylated (Fig. 5A). This supports the notion that FV2_CHO_-specific IgG reactivity reflects PM exposure in South America, as it does in Africa (45), whereas FV2_BIC_-reactive IgG does not. Furthermore, most women with Fc-afucosylated FV2_BIC_-reactive (Fig. 5B) and FV2_CHO_-specific IgG (Fig. 5C)—including the majority of those with marked Fc-afucosylation—had direct evidence of *P. falciparum* infection during their current pregnancy. However, some women without such evidence of exposure to *P. falciparum* also showed Fc-afucosylation of FV2_BIC_-reactive and/or FV2_CHO_-specific IgG (Fig. 5B and C). It is possible that these women had undetected *P. falciparum* infections during their current pregnancy. Alternatively, they might have been exposed to VAR2CSA during a previous pregnancy, as acquired Fc-afucosylation of PfEMP1-specific IgG is a stable phenotype that can be maintained for decades (45, 46).

## DISCUSSION

PfEMP1 is a family of variant *P. falciparum* proteins expressed on the IE surface in a mutually exclusive manner (reviewed in references 3, 4). This means that only one variant is expressed per IE at any given time, although the parasites can switch from expression of one variant to another. The different PfEMP1 antigens mediate IE adhesion to different host vascular receptors, such as CD36 (47), EPCR (48), and ICAM1 (49, 50), to evade IE destruction in the spleen. VAR2CSA is a functionally distinct type of PfEMP1, which exclusively mediates adhesion to CSA, normally only found in the placenta (8). In Africa, VAR2CSA-specific IgG (8) and IgG specifically binding to CSA-adhering IEs (6, 51) are restricted to women who have been exposed to *P. falciparum* parasites during pregnancy. In other words, neither African women who have never been pregnant nor men and children have VAR2CSA-specific IgG, despite marked antibody reactivity to other *P. falciparum* antigens, including non-VAR2CSA PfEMP1 (8, 34). This strongly indicates that VAR2CSA is antigenically distinct from all other *P. falciparum* antigens. However, in 2014, it was reported that IgG reacting with the DBL3X domain, the DBL5ε domain, and the ID1-ID2 minimal binding region of VAR2CSA are common among Colombian men and children (52). These included individuals exposed only to *P. vivax*, the most common malaria parasite in South America, and even some without exposure to malaria parasites at all. The same authors subsequently proposed that their finding was due to IgG induced by exposure to the *P. vivax* merozoite protein PvDBP but cross-reacting with VAR2CSA (38). Indeed, a study done by us but employing a different set of samples yielded very similar results with respect to the prevalence in Colombia of IgG reacting with recombinant VAR2CSA expressed in baculovirus-infected insect cells (FV2_BIC_) (22). However, we also noticed a remarkably discordant difference between levels of FV2_BIC_-reactive and FV2_CHO_-specific IgG (22). Thus, FV2_CHO_-specific IgG was essentially restricted to women exposed to *P. falciparum* infection during pregnancy, whereas FV2_BIC_-reactive IgG was not. Furthermore, only FV2_CHO_-specific IgG levels correlated significantly with IgG reactivity to native VAR2CSA expressed on the IE surface, and there was no significant correlation between IgG reactivity to PvDBP and VAR2CSA (neither correlation was reported in the earlier studies from Colombia and Brazil (38, 52)). Finally, in the earlier study, we found a highly significant correlation between FV2_BIC_- and FV6_BIC_-reactive IgG among Colombian individuals (22). This was unexpected, as FV6_BIC_ corresponds to the full ectodomain of HB3VAR06, which is a non-VAR2CSA-type PfEMP1 unrelated to PM pathogenesis. Rather, it mediates IE rosetting (53), a phenotype implicated in the pathogenesis of severe malaria in children (54). We concluded that FV2_BIC_-reactive IgG is an unreliable indicator of exposure to PM in Colombian individuals and that exposure to *P. vivax* does not appear to induce IgG cross-reactive with native VAR2CSA (22).

In the present study from a geographically distant site in Brazil, we confirm and extend the above findings and conclusions from our earlier study in Colombia. Specifically, we found that FV2_BIC_-reactive IgG was prevalent among Brazilian pregnant women at delivery, whereas FV2_CHO_-specific IgG was not (Fig. 1A and B). Second, levels of FV2_BIC_- and FV2_CHO_-reactive IgG correlated poorly, despite the amino acid identity of the two antigens (Fig. 1C). Third, levels of IgG reacting with the native VAR2CSA on the surface of IEs correlated significantly with levels of FV2_CHO_-specific, but not with FV2_BIC_-reactive IgG (Fig. 2), as observed previously (22, 55). Fourth, FV2_BIC_-reactive IgG was found at similar levels among women exposed to *P. falciparum* and *P. vivax* infection during their current pregnancy (Fig. 3A), whereas levels of FV2_CHO_-specific IgG above negative cutoff were largely restricted to women exposed to *P. falciparum* (Fig. 3B). Fifth, levels of FV2_BIC_-reactive IgG did not correlate with levels of PvDBP-specific IgG (Fig. 4). Finally, the FV2_BIC_-reactive IgG observed in the present study was largely fully Fc-fucosylated (Fig. 5), suggesting that it was not acquired because of exposure to VAR2CSA-positive IEs, and importantly that it may have limited protective efficacy against PM (because of its inability to induce antibody-dependent cellular cytotoxicity [ADCC]) (31).

Potentially, VAR2CSA-specific IgG can protect against PM by inhibiting VAR2CSA-specific IE sequestration of IEs in the placenta (neutralization) and/or by opsonizing them for phagocytosis (56, 57) or destruction by ADCC (31, 58). Although a neutralizing VAR2CSA-specific IgG response is often assumed to be decisive (23), the evidence underpinning this assertion remains woefully equivocal (reviewed in reference 59). Indeed, several studies indicate that clinical protection from *P. falciparum* malaria reflects the combined impact of multiple effector functions and that opsonization by IgG for Fc-dependent destruction of parasites and IEs is more important than has previously been assumed (46, 60–62). Natural killer cell destruction of IgG-opsonized IEs by ADCC is one example (58). This response is activated through FcγRIIIa, which has low affinity for IgG, unless the opsonizing IgG is Fc-afucosylated (63). It is therefore noteworthy that PfEMP1-specific IgG is markedly Fc-afucosylated (31, 45, 46). Indeed, the degree of Fc-afucosylation of VAR2CSA-specific IgG appears to be a stronger determinant of clinical protection against PM than levels of VAR2CSA-specific IgG *per se* and levels of IE adhesion-inhibitory IgG (31).

It remains unclear why levels of plasma IgG to otherwise identical recombinant PfEMP1 expressed in baculovirus-infected insect cells and CHO cells are discordant among Colombian and Brazilian individuals, as this appears not to be the case among Africans (22, 64). It is also unknown whether responses to other recombinant antigens expressed in baculovirus-infected insect cells are similarly affected, and whether the phenomenon is confined to South America. These are issues beyond the scope of the current investigation, but relevant in many contexts. Until they are resolved, caution should be exerted.

## SUPPLEMENTAL MATERIAL

Supplemental material iai.00516-25-s0001.pdf Table S1; Fig. S1 and S2.

ASM does not own the copyrights to Supplemental Material that may be linked to, or accessed through, an article. The authors have granted ASM a non-exclusive, world-wide license to publish the Supplemental Material files. Please contact the corresponding author directly for reuse.
