# Supplementary material for: VAR2CSA-reactive IgG in Brazilian women exposed to Plasmodium falciparum or P. vivax infection during pregnancy
Source: Infect Immun. 2025 Oct 31;93(12):e00516-25. doi: 10.1128/iai.00516-25 (PMC12707141; doi:10.1128/iai.00516-25)

Supplementary information (Santos *et al.*)

Supplementary Table 1

Correlations (Spearman's rank correlation) between IgG responses to the antigens studied

	Antigen	GLURP	PvDBP
All women (58/380) [a]	$FV2_{BIC}$	0.07 (p=0.60) / 0.27 (p=0.001)	0.12 (p=0.36) / 0.21 (p=0.01)
	$FV2_{CHO}$	0.34 (p=0.01) / 0.24 (p=0.002)	0.35 (p=0.01) / 0.45 (p<0.001)
P. falciparum-infected (18/70)	$FV2_{BIC}$	-0.19 (p=0.46) / 0.09 (p=0.45)	-0.34 (p=0.17) / 0.12 (p=0.34)
	$FV2_{CHO}$	0.15 (p=0.55) / 0.29 (p=0.02)	0.27 p=0.28) / 0.19 (p=0.11)
P. vivax-infected (26/150)	$FV2_{BIC}$	-0.14 (p=0.50) / 0.20 (p=0.02)	0.23 (p=0.25) / 0.11 (p=0.19)
	$FV2_{CHO}$	0.09 (p=0.66) / 0.28 (p=0.001)	0.15 (p=0.46) / 0.17 (p=0.04)
Non-infected (14/160)	$FV2_{BIC}$	-0.44 (p=0.12) / 0.27 p=0.001)	-0.17 (p=0.56) / 0.21 (p=0.01)
	$FV2_{CHO}$	0.35 (p=0.21) / 0.24 p=0.002)	0.69 (p=0.01) / 0.45 (p<0.001)

[a] Values before slashes include only women with levels of $FV2_{BIC}$-reactive IgG above negative cutoff, while values after the slash include all women.

Supplementary Fig. 1

Gating strategy for IgG binding to infected erythrocytes (IEs). Purified late-stage IEs were labeled with plasma followed by FITC-conjugated rabbit anti-human IgG and Hoechst 33342. (A) IEs were gated using Hoechst vs. FSC-A to select the late-stage IEs (Hoechst positive). (B) The gated late stage-IE population was used to plot histograms of FITC-A and to determine the population of IEs binding IgG. Representative flow cytometry histograms of IEs labelled with immune plasma from Brazilian women without evidence of infection, from Brazilian women with *P. vivax* or *P. falciparum* infection during the current pregnancy, and (for comparison) with pooled plasma from PM-exposed women from Ghana are shown (gray shading). IgG labelling with non-immune IgG is also shown (no shading).

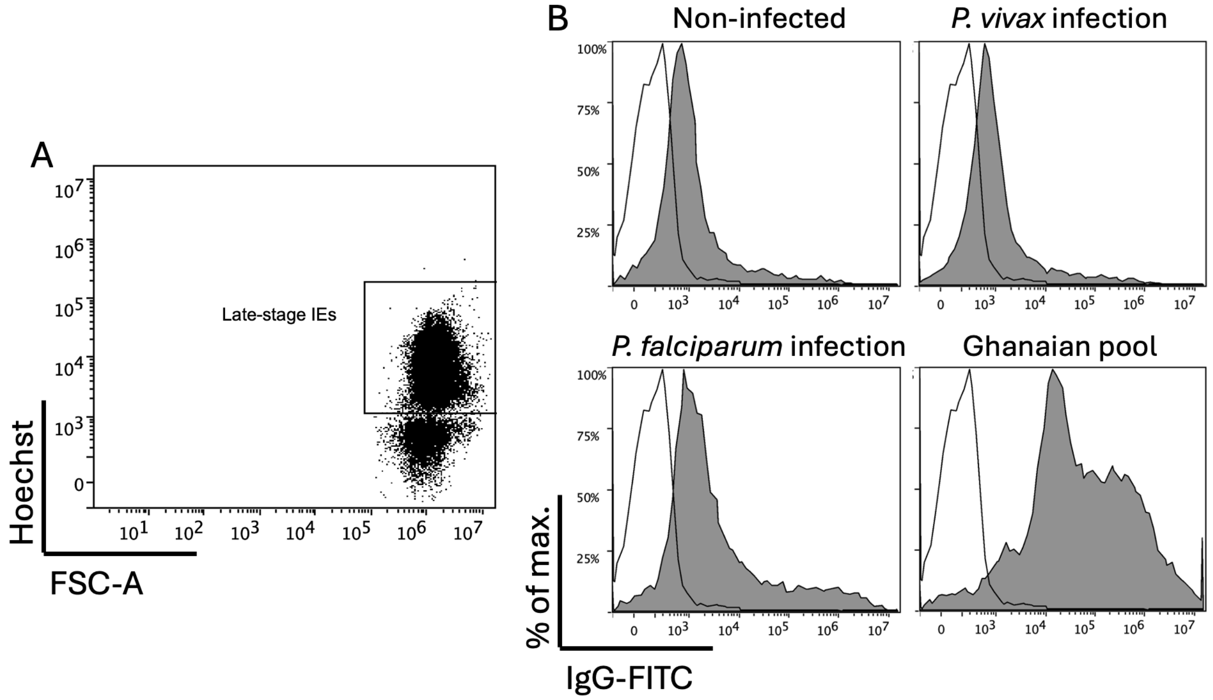

Supplementary Fig. 2

Exposure to malaria parasites during pregnancy and IgG responses to GLURP at delivery among women with documented *P. falciparum* (triangles) or *P. vivax* infection (squares) or without evidence of infection (diamonds) during the current pregnancy. Individual data points, medians, 95% confidence intervals, negative cutoff (shading), and results of Kruskal-Wallis test followed by Dunn's test are shown. The number of data points above negative cutoff followed by the total number of data points are shown in brackets.

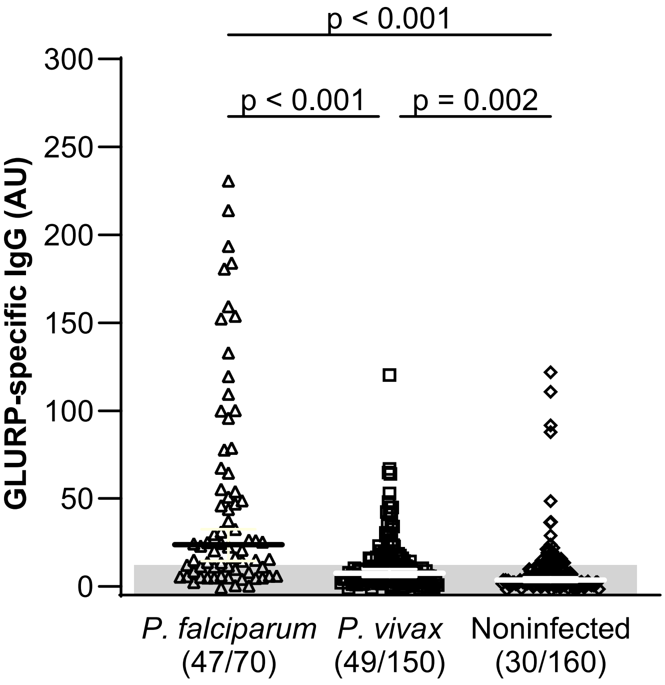

Supplement: Supplemental material — Table S1; Fig. S1 and S2. [file iai.00516-25-s0001.pdf]
